# Comparative Analysis of the Mitochondrial Genome Sequences of *Diaporthe longicolla* (syn. *Phomopsis longicolla*) Isolates Causing Phomopsis Seed Decay in Soybean

**DOI:** 10.3390/jof10080570

**Published:** 2024-08-13

**Authors:** Shuxian Li, Xiaojun Hu, Qijian Song

**Affiliations:** 1 United States Department of Agriculture, Agricultural Research Service (USDA, ARS), Crop Genetics Research Unit, 141 Experiment Station Rd., Stoneville, MS 38776, USA; 2 USDA, Animal and Plant Health Inspection Service (APHIS), Plant Protection and Quarantine (PPQ), Plant Germplasm Quarantine Program (PGQP), Beltsville, MD 20708, USA; xiaojun.hu@usda.gov; 3 USDA, ARS, Soybean Genomics and Improvement Laboratory, Beltsville Agriculture Research Center, Beltsville, MD 20705, USA; qijian.song@usda.gov

**Keywords:** *Diaporthe longicolla*, mitochondrial genome, Phomopsis seed decay, soybean pathogen

## Abstract

*Diaporthe longicolla* (syn. *Phomopsis longicolla*) is an important seed-borne fungal pathogen and the primary cause of Phomopsis seed decay (PSD) in soybean. PSD is one of the most devastating seed diseases, reducing soybean seed quality and yield worldwide. As part of a genome sequencing project on the fungal *Diaporthe–Phomopsis* complex, draft genomes of eight *D. longicolla* isolates were sequenced and assembled. Sequences of mitochondrial genomes were extracted and analyzed. The circular mitochondrial genomes ranged from 52,534 bp to 58,280 bp long, with a mean GC content of 34%. A total of 14 core protein-coding genes, 23 tRNA, and 2 rRNA genes were identified. Introns were detected in the genes of *atp6*, *cob*, *cox1*, *cox2*, *cox3*, *nad1*, *nad2*, *nad5*, and *rnl*. Three isolates (PL7, PL10, and PL185E) had more introns than other isolates. Approximately 6.4% of the mitochondrial genomes consist of repetitive elements. Moreover, 48 single-nucleotide polymorphisms (SNPs) and were identified. The mitochondrial genome sequences of *D. longicolla* will be useful to further study the molecular basis of seed-borne pathogens causing seed diseases, investigate genetic variation among isolates, and develop improved control strategies for Phomopsis seed decay of soybean.

## 1. Introduction

The seed-borne fungal pathogen *Diaporthe longicolla* (Hobbs) J. M. Santos, Vrandecic & A. J. L. Phillips (syn. *Phomopsis longicolla* T. W. Hobbs) is the primary cause of Phomopsis seed decay (PSD) in soybean [*Glycine max* (L.) Merrill] [1,2,3,4,5,6]. The PSD-causing pathogen was first identified as *Phomopsis longicolla* in 1985 [1]. Later, in 2011, the fungus was renamed *Diaporthe longicolla* [6]. However, the disease is still retained as Phomopsis seed decay although the fungus name has been changed.

PSD is one of the most destructive seed diseases, reducing soybean seed quality and yield worldwide. The most charactered symptoms of PSD include seed discoloration with cracked seed coats. The entire seed surface can be moldy and have a chalk-white color if soybean seeds are severely infected with *D. longicolla.* In some cases, *D. longicolla*-infected soybean seeds did not have visible symptoms [7,8]. Moreover, seed compositions could be adversely altered or reduced by the infection of *D. longicolla* [2]. In the mid-southern region of the United States, especially under the humid and warm conditions that favor the colonization of the pathogen, PSD has significantly economic impact on soybean production [8,9,10].

Breeding for host genetic resistance is one of the most effective means to manage PSD in an environmentally friendly approach [11,12,13]. In the past decades, extensive research efforts have been made to screen soybean germplasm and cultivars to identify sources of resistance to PSD [9,10,14,15]. However, development of resistant cultivars may depend on the variability of the pathogen, including isolate aggressiveness. Knowledge about the variability of the pathogen is crucial for understanding the pathogen population, which will also be important for selection of isolates for developing soybean lines with broad-based resistance to Phomopsis seed decay and high seed quality. Differences between *D. longicolla* isolates in their ability for infecting soybean have been reported. For an example, in evaluating the aggressiveness of 48 isolates of *D. longicolla* from different geographic origins, which included 35 *D. longicolla* isolates from soybean in eight states in the United States, 2 *D. longicolla* isolates from velvetleaf in Illinois [16], and 11 other *Phomopsis* spp. isolates from other hosts in four states in the United States as well as Canada and Costa Rica, it was found that there were significant differences in stem lesion length of a susceptible cultivar, Williams 82, after inoculation with different isolates, and the most aggressiveness isolates were identified [4]. Similar results of difference in isolate aggressiveness on soybean were also reported in another study [17].

To facilitate investigation of the genomic basis of the pathogenicity and variability of isolates and to understand the mechanism of disease development, as part of a genome-sequencing project on the fungal *Diaporthe–Phomopsis* complex, the genome of a *D. longicolla* isolate (MSPL10-6) from Mississippi, USA, was sequenced and analyzed [18]. Preliminary study on its mitochondrial genome was conducted [19]. It is well known that mitochondria are semi-autonomous organelles that have their own genetic material (DNA) and are capable of synthesizing proteins required for their functioning, existing in most eukaryotic cells. Mitochondria play crucial roles in essential energy production, respiratory metabolism, cell growth, and many other essential cellular processes [20]. Because of the relatively small size, high copy number, and high mutation rate, sequences of fungal mitochondrial genome (mitogenome) have been used as valuable tools to develop molecular markers for species identification and characterization [21,22]. However, the mitochondrial genome of different *D. longicolla* isolates from different geographic origins has not been well studied. Although variation of different isolates in aggressiveness and virulence has been reported [4,17], the genomic variation is unclear. Repetitive and transposable elements in mitochondrial genomes could play an important role in fungal diversity [23].

As a continuing effort to explore the mitochondrial genome of *D. longicolla*, eight isolates from different states in the U.S. (Table 1) were sequenced and analyzed in this study. The objectives of this study were (1) to study the gene content and organization of the mitochondrial genome of *D. longicolla*; (2) to compare differences in the occurrence and distribution of mobile genetic elements, such as the intron’s types and numbers, as well as transposable elements in different *D. longicolla* isolates; and (3) to identify repetitive elements in the mitochondrial genome. The mitochondrial genome sequences of *D. longicolla* will be useful to further study the molecular basis of seed-borne pathogens causing seed disease in soybean, investigate genetic variation among isolates from different geographic origins, and develop improved control strategies for Phomopsis seed decay of soybean.

## 2. Material and Method

### 2.1. Fungal Isolates and DNA Extraction

Eight isolates of *D. longicolla* originating from six states of USA were used in this study, including the type culture of TWH P74 (ATCC 60325) obtained from the American Type Culture Collection in 2010 (Table 1). Total genomic DNA of each isolate was extracted using a Qiagen DNeasy Plant Mini Kit (Qiagen Inc., Valencia, CA, USA) after culturing in potato dextrose broth (Difico Laboratories, Detroit, MI, USA) in an incubator statically for 4 days under 12-hour light and dark cycles at 24 °C. The quality of DNA was assessed by 1% agarose gel electrophoresis, and DNA was quantified using a Nanodrop 1000 (NanoDrop Technologies, Inc., Wilmington, DE, USA) and a Qubit v.2.0 fluorometer (Fisher Scientific, Waltham, MA, USA).

### 2.2. Genome Sequencing and Mitochondrial DNA Assembly

Genome sequencing was carried out either at the Genomics Core Facility, Purdue University at West Lafayette, Indiana, using the Illumina HiSeq 2500 (Illumina, Inc., San Diego, CA, USA) sequencer (for isolates MSPL10-6, TWH P74, and PL185E), as reported previously [18], or Novogene (for isolates PL1, PL6, PL7, PL10, and PL11) using HiSeq PE 150 (Illumina, Inc., San Diego, CA, USA). The *D. longicolla* genomes were assembled using the Spades program (v3.15.4) [24]. Mitochondrial contigs were picked out from assembly results by comparing with the published mitochondrial DNA of *D. longicolla* isolates at GenBank (accessions KP 137411.1 and MT 527962) using BLASTX [25]. Then, the reads mapped to those contigs were assembled again, using Spades (v3.15.4), into a circular mitochondrial genome.

### 2.3. Gene Annotation and Analysis

Gene annotation (including intron identification) was conducted using the online program MFannot (https://megasun.bch.umontreal.ca/apps/mfannot/, accessed on 1 November 2023). The intron in the nad2 gene of isolates (PL7, PL10, PL11, and PL185E) was identified in the alignment of the mitochondrial genome sequences *D. longicolla*, which was performed by MAFFT (v7.490) [26] with an automatic algorithm (parameter: mafft—auto). Its conserved intron domain (double LAGLIDADG_1) was searched using the online program Pfam (http://pfam.xfam.org/, accessed on 1 November 2023). The intron type was determined by the online program RNAweasel (https://megasun.bch.umontreal.ca/apps/rnaweasel/, accessed on 1 November 2023). The intron–exon border was identified by viewing the alignment using BioEdit (v7.2.0) [27].

The physical maps of the mitochondrial genome of eight *D. longicolla* isolates were drawn using the program Circos (v0.69-9). The secondary structure of tRNA genes was predicted and drawn using the MITOS2 web server (https://usegalaxy.org/root?tool_id=toolshed.g2.bx.psu.edu%2Frepos%2Fiuc%2Fmitos2%2Fmitos2%2F2.1.3%20galaxy0, accessed on 1 November 2023). The codon usage was calculated by Sequence Manipulation Suite (https://www.bioinformatics.org/sms2/codon_usage.html, accessed on 1 November 2023) with genetic code 4.

### 2.4. Identification of Repetitive Elements

Four methods were used to analyze repetitive elements in the mitochondrial genomes of eight *D. longicolla* isolates: (1) a BLASTn search [28] of the whole mitochondrial genome against itself to determine if there was intra-genomic duplication of large fragments at an E value of <10^−10^; (2) the online program Tandem Repeat Finder, with the default parameter to detect tandem repeats (>10 bp in length) [29]; (3) the REPuter tool (https://bibiserv.cebitec.uni-bielefeld.de/reputer/, accessed on 1 November 2023) to identify forward, reverse, complemented, and palindromic repeats [30]; and (4) the EMBOSS (v6.6.0) Suite to identify inverted repeats [31].

Simple sequence repeats (SSRs) with di- and tri-nucleotide repeats (> 5 bp) were identified with the Perl script MISA tool [32]. The sequences were aligned by Muscle [33] in the Mega6 program [34], and the aligned sequence file was imported into the DNASP6 program [35,36] for identification of single-nucleotide polymorphisms (SNPs). SNPs were verified by Sanger sequencing the PCR fragments containing the SNPs.

## 3. Results

### 3.1. General Feature of D. longicolla Mitochondrial Genome

**Figure 1:**
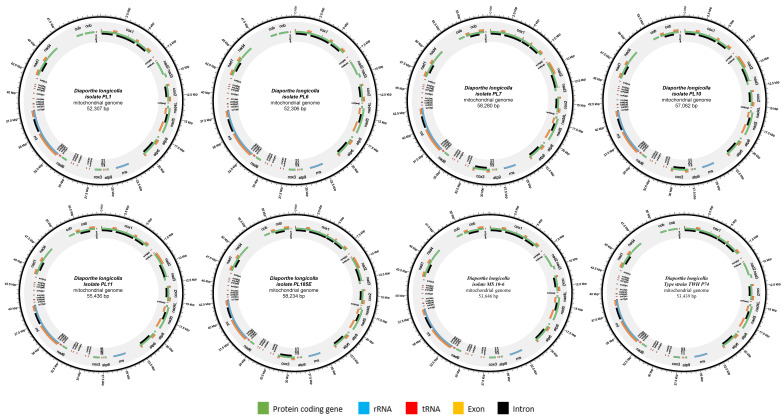
Circular maps of the mitochondrial genomes of eight *Diaporthe longicolla* isolates.

The mitochondrial genomes of eight *D. longicolla* isolates were assembled into a single circular, double-stranded DNA molecule (Figure 1). The sizes of the mitochondrial genomes ranged from 52,534 bp to 58,280 bp. Isolate PL6 had the smallest size of 52,306 bp, while isolate PL185E had the largest size of 58,234 bp (Table 2). The isolates (PL7, PL10, and PL185E) had more introns than the other isolates. The mean GC content was 34%. The GC skew was positive (0.10), while the AT skew was slightly negative (−0.03), indicating that there was no significant strand-specific bias of nucleotide composition in the mitochondrial genome of *D. longicolla.*

A total of 14 core mitochondrial protein-coding genes were identified. In addition to the core protein-coding genes, four and five free-standing open-reading frames of unknown function (uORFs) were annotated from two and six isolates, respectively (Table 2). The mitochondrial genomes of *D. longicolla* isolates also contained 23 tRNA genes, except isolate PL185E, which had 22 tRNA genes. The mitochondrial genomes of *D. longicolla* also contained two rRNA genes, a large subunit ribosomal RNA gene (*rnl*), and a small subunit of ribosomal RNA gene (*rns*) as well as a gene coding the 40S ribosomal protein S3 (*rps3*).

### 3.2. Protein-Coding Genes and Codon Usage

The 14 conserved protein-coding genes encoded three subunits of the ATP-synthase (*atp6*, *atp8*, and *apt9*), seven subunits of electron transport chain of complex I (*nad1*, *nad2*, *nad3*, *nad4*, *nad4L*, *nad5*, and *nad6*), three subunits of complex III (*cox1*, *cox2*, and *cox3*), and one subunit of complex IV (*cob*). Of the 14 protein-coding genes identified, 9 contained introns, which were located at the *atp6*, *cob*, *cox1*, *nad2*, *cox2*, *cox3*, *nad1*, *nad5*, and *rnl* genes (Table 3).

The *cox1* gene had the highest number of intron sequences (four in five isolates and five in three isolates). The total number of introns ranged from 9 to 13 among isolates. Isolates PL7 and PL185E had the highest number of 13 introns, and isolate PL10 had 12, while isolates PL1 and PL6 had nine introns. Both MSPL10-6 and TWH P74 had 10 introns, respectively. Isolates MSPL10-6, TWH P74, PL1, and PL6 did not have an intron in *nad2* and *cox3* genes (Table 3). Additional information about introns identified in the mitochondrial genome of *D. longicolla* isolates is shown in Table 4.

**Figure 2:**
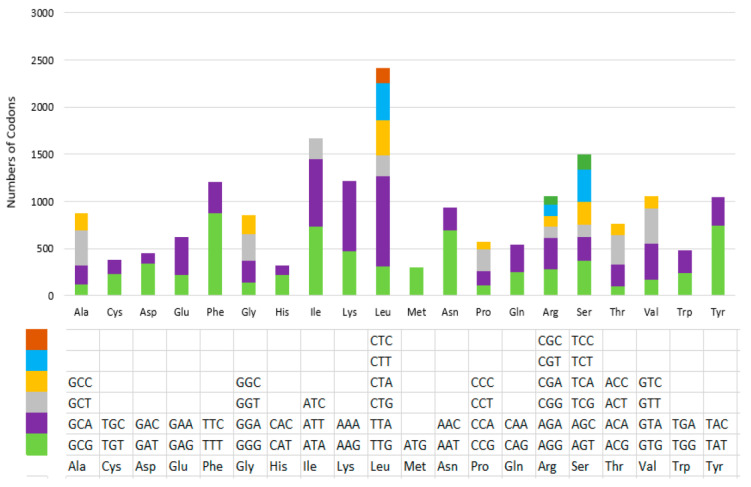
Codon usage in the mitochondrial genome of *Diaporthe longicolla* isolate PL185E. Codon families are plotted on the X axis and represented by different color patches. Frequency of codon usage is plotted on the Y axis. The codon usage was calculated by the Sequence Manipulation Suite (https://www.bioinformatics.org/sms2/codon_usage.html, accessed on 1 November 2023) with genetic code 4.

The most common start codon for the 14 protein-coding genes was ATG, while AAA, ACA, ACG, ATA, ATG, TTA, and TTT were also utilized. The most frequent stop codon was TAA, but TAG was also used. Codon usages in the isolate of PL185E, as an example, are presented in Figure 2.

The gene order for the *D. longicolla* mitochondrial genome, starting with *cox1*, was *cox1*–*nad2*–*nad3*–*cox2*–*nad4L*–*nad5*–*atp8*–*apt6*–*rns*–*apt9*–*cox3*–*nad6*–*rnl*–*rps3*–*nad1*–*nad4*–*cob-1*–*cob-2*. In comparison of gene order across eight isolates of *D. longicolla*, no gene rearrangement was found in those isolates.

### 3.3. Transfer RNA and Ribosomal RNA Genes

**Figure 3:**
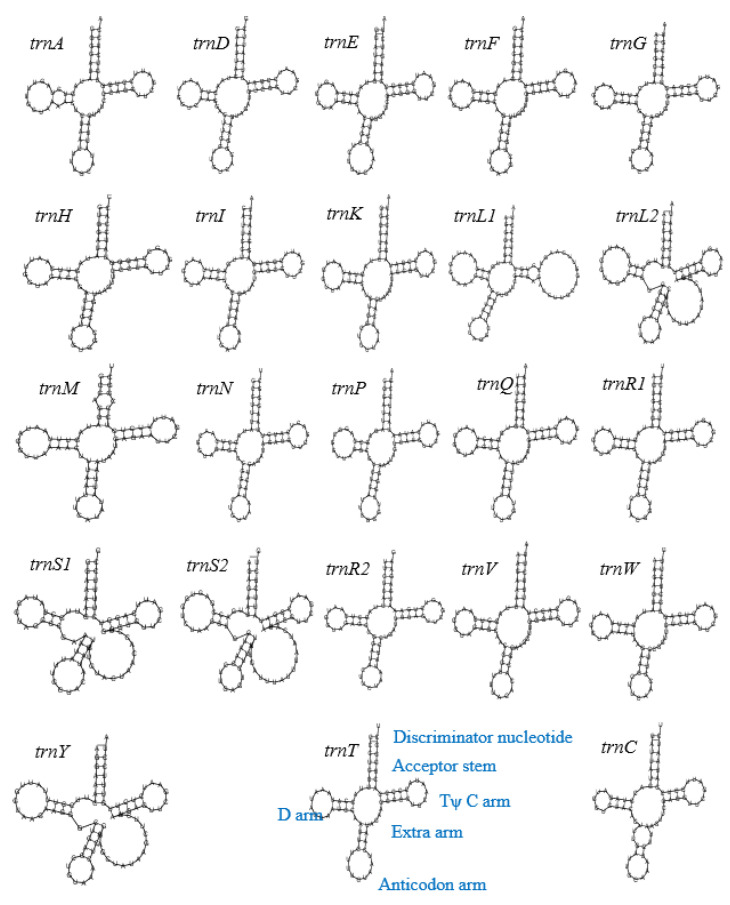
Putative secondary structures of the 23 tRNA genes from PL mitochondrial genome. The tRNAs are labeled with the abbreviations od their corresponding ammino acids. The tRNA arms are illustrated as from *trnT*. The map of tRNA structures was drawn using the MITOS web server (http://mitos.bioinf.uni-leipzig.de/index.py, accessed on 1 November 2023).

A total of 23 transfer RNA (tRNA) genes were identified in the mitochondrial genomes of *D. longicolla* coding for 20 amino acids, except for isolate PL185E, which had 22 tRNA genes coding for 19 amino acids. The length of the tRNA genes ranged from 69 bp (*trnF*) to 85 bp (*trnS*). Two tRNA genes (*trnM* and *trnF*) were duplicated. The *trnM* (cat) had three copies in all eight *D. longicolla* isolates. Isolate PL185E contained two copies of *trnF*: One was *trnC* (gca) as other isolates had, and another was *trnF* (gaa). Most of the tRNA genes were located between *rnl* and *nad1*. No introns were found in tRNA genes. The tRNA molecules can fold into a common cloverleaf secondary structure comprised of the acceptor stem, D-loop, anticodon, and TψC loop (Figure 3). There was a large loop structure on the extra arm of *trn*L2, *trn*S1, *trn*S2, and *trn*Y. In *trn*C, there were two loops on the anticodon arm, while other tRNA genes had only one loop on the anticodon arm. Their graphic depictions are shown in Figure 3.

There were two ribosomal RNA (rRNA) genes in the mitochondrial genome of *D. longicolla* (*rnl* and *rns*). The *rnl* was 6050 bp in length, which was interrupted by an intron with a length of 2123 bp. The *rns* was 1637 bp, without an intron. The *rnl* was located between *trn*P and *trn*T, while the *rns* was placed between *apt6* and *atp9*.

### 3.4. Repetitive Elements

**Figure 4:**
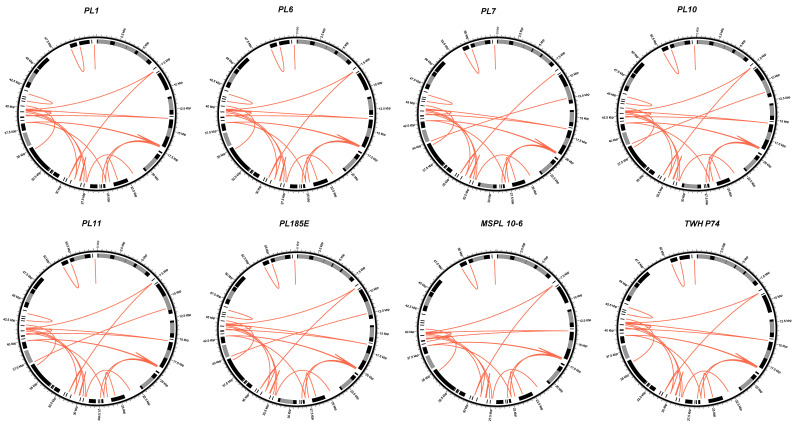
Dispersed and inverted repeat sequences in *Diaporthe longicolla* mitochondrial genome. Colors: black, conserved protein-coding, rRNA and tRNA genes; grey, introns; white, intergenic regions. Red lines connect regions of significant (E-value < 1 × 10^−10^) nucleotide sequence similarity.

A sequence BLASTn search against itself of each mitochondrial genome revealed 28 to 32 repetitive sequences among eight isolates, accounting for approximately 6.4% of the *D. longicolla* mitochondrial genomes. The size of repeats ranged from 33 bp to 336 bp (Table S1). Analysis with the Tandem Repeat Finder (TRF) program identified seven or eight tandem repeats with consensus sizes from 8 to 37 bp in each isolate. There were seven repeat motifs in isolates PL1, PL6, MSPL10-6, and TWH P74 (ATCC 60325) and eight repeat motifs in isolates PL7, PL10, PL11, and PL185E (Table S2). In addition, there was a tandem repeat element in the *nad2* intron in the genomes of PL7, PL10, PL11, and PL185E. Using the REPuter program, 30 or 31 forward and 19 or 20 palindromic repeats were detected among isolates. (Table S3). A total of 20–22 short, inverted repeats with a size range from 17 to 54 bp were identified by the EMBOSS program (Table S4). Dispersed and inverted repeat sequences of eight isolates are shown in Figure 4. No reverse repeat sequences were identified.

Using Perl script MISA [32] to search the simple sequence repeats (SSRs) with di-, tri-, and tetranucleotide repeat units larger than 4 bp in the mitochondrial genome sequences of eight *D. longicolla* isolates, 24 SSRs with di- and tri-nucleotide repeats (>5 bp) were identified, 16 of which had AT motifs, and 8 SSRs had ATT motifs. Each mitochondrial sequence contains two SSRs with AT motifs and one SSR with ATT motifs. There was no polymorphism in these SSRs found among the isolates. However, single-nucleotide polymorphisms (SNPs) were detected. There were 48 polymorphic sites, including 20 singleton variable sites and 28 parsimony informative sites (Table 5), when the mitochondrial genome sequences were aligned by Muscle [33] in the Mega6 program [34]. Of eight isolates studied, seven had the same SNP at 14 sites. Isolate MSPL 10-6, originating from Mississippi, had more SNPs than all other isolates including two more isolates from Mississippi.

## 4. Discussion

With the advancement of high-throughput sequencing technologies and facilities, the feasibility of mitochondrial genome sequences has been increasing. At present, at least 4201 fungal mitochondrial genome sequences are available at GenBank (https://www.ncbi.nlm.nih.gov/nuccore/?term=fungi%5BOrganism%5D+AND+mitochondrion%5Bfilter%5D+AND+complete%5Btitle%5D+AND+ddbj_embl_genbank%5Bfilter%5D+NOT+unverified, accessed on 28 January 2024). However, information about the mitochondrial genome of the fungal pathogens that cause soybean diseases is still limited. Most of the reported studies focused on either one specific species or compared two or more closely related species using only one fungal isolate or strain of each, such as the isolate Pg-21 of *Coniothyrium glycines* that causes red leaf blotch of soybean [37] and two *Phakopsora* species, namely *P. pachyrhizi* (isolate Taiwan 72-1) and *P. meibomiae* (an isolate from Puerto Rico), the causal agents of soybean rust [38].

Our present study was the first report of analysis of the mitochondrial genome sequences of eight *D. longicolla* isolates collected from different states in the U.S. Eight assembled mitochondrial genomes were compared to reveal a general structure, gene content, repetitive and transposable elements, and a possible pattern of variation. Like most other fungi, the mitochondrial genomes of eight *D. longicolla* isolates were assembled into a single circular, double-stranded DNA molecule. It contains a small set of conserved genes, including 14 protein-coding genes, 22 or 23 tRNA genes, as well as the small and large ribosomal RNA genes.

In the mitochondrial genomes of fungi, duplicated copies of conserved protein-coding genes are quite common. For example, there was an extra copy of *atp9* in the mitochondrial genome of the phytopathogenic fungus *Sclerotinia borealis* [39]. The gene duplication was also reported in *Botryotinia fuckeliana* [39], *Phlebia radiata* [40], as well as many other fungal species. In our present study, the mitochondrial genomes of all eight *D. longicolla* isolates contained a truncated extra copy of *cob*, which was 160 bp of exon 1 in *cob*. Besides the conserved protein-coding genes, the tRNA gene *trnM* (cat) had three copies in all eight *D. longicolla* isolates, and isolate PL185E also contained two copies of *trnF*. The cause of those gene duplication and their impact on the function of mitochondria are not well understood and remain for further study.

There were remarkable size variations of mitochondrial genomes in numerous fungi [41]. The small mitochondrial genomes included those of about 18.84 kp in *Hanseniaspora uvarum* [42], whereas the larger mitochondrial genomes in fungi were more than 200 kb, such as those of 203 kp in *Sclerotinia borealis* [39] and 272.2 kb in *Morchella importuna* [43]. Results from the analysis of the mitochondrial genomes of nine *Aspergillus* and *Penicillium* species indicated that the number of introns and their length difference represent one of the major factors contributing to variations in the mitochondrial genome size of fungal species [44]. Analysis of 11 complete mitogenomes of *Polyporales* species revealed the great variations in intron distribution and content [45]. In our study, the sizes of the mitochondrial genomes of eight *D. longicolla* isolates ranged from 52,534 bp to 58,280 bp. Three isolates (PL7, PL10, and PL185E) had more introns and larger size than the other isolates. More research should be conducted to address if the differences in the mitochondrial genome size relate to the genetic diversity and evolutionary dynamics of this fungal species.

The numbers of intron in mitochondrial genomes vary in different fungal species [20]. In general, mitochondrial introns in fungi can be mainly classified into two groups (groups I and II) according to their conserved RNA secondary structures [46,47]. Group I introns are large self-splicing ribozymes. They catalyze their own excision from mRNA, tRNA, and rRNA precursors in a wide range of organisms and are rich in fungi [48]. Group I introns are further divided into subgroups such as IA, IA3, IB, IC1, IC2, and ID based on phylogenetic analyses [46]. In general, group I introns are prevalent in fungal mitochondrial genes with greater association for genes, e.g., *cox1*, *cob*, and *rnl*, while group II introns are predominant in plant mitochondrial genomes [46]. The mitochondrial *cox1* gene has been reported to be the richest in group I introns [49]. In contrast, the *Fusarium oxysporum* species complex (FOSC) did not possess any introns in its *cox1* gene [50]. In the case of *D. longicolla*, most introns located in the *cox1* gene were all IB type, while IA introns were found in *cob*, *cox3, nad2*, and *rnl*; IB introns were found in *cox2*, *nad1*, *nad3*, and *nad5*; and IC introns were found in in *Atp6* genes. These *D. longicolla* introns carried either LAGLIDADG or GIY-YIG homing endonuclease genes, which have been reported to facilitate the movement of introns into previously intron-less genes or certain regions, resulting in enlargement of the mitochondrial genome size [46]. Although introns are often found in mitochondrial genomes, we are still uncertain about their origins and the modes of transmission in fungi.

Although high variability of mitochondrial gene order among fungi has been reported [51,52,53], some closely related fungal groups appeared to be conserved. The order of protein-coding genes and tRNA was identical in two related soybean rust pathogens, *Phakopsora pachyrhizi* and *P. meibomiae* [38]. Results from the analysis of the distribution of mitochondrial protein-coding genes indicated no significant differences in gene order among *Hypocreales* species [54]. Comparison of *Verticillium nonalfalfae* and the closely related *V. dahliae* revealed a conserved gene order in their mitochondrial genomes [55]. In our present study, the gene order of all eight *D. longicolla* isolates was the same. No gene rearrangement was found among isolates. It is interesting to see such consistency of the gene order in their mitochondrial genomes. The absence of gene rearrangement among the *D. longicolla* isolates in their mitochondrial genomes contributes to our understanding of their genetic stability and lays the foundation for further investigation of their evolutionary patterns.

One of the most noticeable features of the fungal genome is the presence of repetitive elements. Generally, over 30% repetitive DNA was found in fungal Zygomycota, whereas more than 5% was reported in ascomycete and basidiomycetes fungi [56]. Results from analysis of a potato fungal pathogen *Rhizoctonia solani* AG-3 revealed that one-third of the mitochondrial genome was occupied by interspersed repeats, which included at least three types: short interspersed palindromic sequences < 40 bp, mid-length (50–95 bp) sequences, and longer elements (>100–963 bp) [57]. In our present study, approximately 6.4% of the *D. longicolla* mitochondrial genomes contained different types of repetitive sequences.

Simple sequence repeats (SSRs) have been considered as a valuable source of genetic markers and are widely used in population genetics, genetic diversity, fingerprinting, and forensic analysis in many organisms due to their abundance and inherent potential for variation [58]. Although there was no polymorphism in simple sequence repeats (SSRs) among the *D. longicolla* isolates, the SSRs identified in this study could be used to develop molecular markers to distinguish *D. longicolla* from other closely related fungal species, especially those in the *Diaporthe–Phomopsis* complex causing soybean diseases as well as *D. nobilis*, an alpine pathogen, which is close to *D. longicolla* based on the analysis of the mitochondrial genome sequences [59]. It is well known that single-nucleotide polymorphisms (SNPs) can be used to characterized similarities and differences within a species of different isolates or between different species [60,61,62]. Characterization of the SNP and structural variations in the mitochondrial genomes of a fungal pathogen *Tilletia indica* causing the disease Karnal bunt in wheat and its closely related species led to developing a simple and high-throughput diagnostic assay [63]. Results from analysis of the mitochondrial genomes of closely related *Fusarium culmorum* and *F. graminearum* demonstrated that the diversity of mobile genetic elements was the main distinctive trait for diagnostic purposes of Fusaria [64,65]. In our present study, 48 SNPs were detected in the mitochondrial genome sequences of eight *D. longicolla* isolates. This information can be utilized to develop molecular markers for the detection and identification of *D. longicolla* in soybean, especially those soybean seeds that have been infected by *D. longicolla* but do not show visible PSD symptoms.

It is well known that the occurrence and severity of plant diseases result from three factors: plant host, pathogen, and environment. This is called the “Disease Triangle” [66], a fundamental concept for disease causation and management. These three factors interact with each other. If any one of the three factors is missing, the “triangle” is not complete, and disease will not occur. The pathogen, *D. longicolla*, is one of the three factors in the PSD disease “triangle”. As the first step toward to disentangling the pathogenicity mechanism of *D. longicolla* causing PSD in soybean, we analyzed the mitochondrial genome sequences of eight *D. longicolla* isolates. This research was one of our efforts to investigate how genetic variations of the pathogen influence PSD development. Identification of genetic factors/genes associated with the pathogenicity of *D. longicolla* through a whole-genome analysis is underway. 

Together, the results of the present study on the comparative analysis of the mitochondrial genome sequences of *D. longicolla* isolates will be useful to further study the molecular basis of seed-borne pathogens causing seed diseases, investigate genetic variation among isolates, and develop improved control strategies for Phomopsis seed decay of soybean.

## Figures and Tables

**Table 1:** A list of *Diaporthe longicolla* (syn. *Phomopsis longicolla*) isolates used in this study and their geographic origins in the U.S. and the year isolated from soybean.

Isolate	Geographic Origin	Year Isolated
PL1	Illinois	2016
PL6	Arkansas	2009
PL7	Missouri	2009
PL10	Mississippi	2016
PL11	Maryland	2016
PL185E	Mississippi	2015
MSPL10-6	Mississippi	2010
TWH P74 ^a^	Ohio	1983

^a^ Type strain TWH P74 (ATCC 60325) was obtained from the American Type Culture Collection (ATCC) in 2010.

**Table 2:** General features of the mitochondrial genome of *Diaporthe longicolla* (syn. *Phomopsis longicolla*).

Isolate	Genome Size (bp)	GC (%)	Number of Conserved Protein-Coding Genes	Number of rRNA	Number of tRNA	Number of Introns	Intronic ORFs	Free-Standing ORFs	Total ORFs
PL1	52,534	34.6	14	2	23	9	8	5	13
PL6	52,306	34.5	14	2	23	9	9	5	14
PL7	58,280	34.1	14	2	23	13	12	4	16
PL10	57,062	34.2	14	2	23	12	10	5	15
PL11	55,837	34.3	14	2	23	11	10	5	15
PL185E	58,234	34.1	14	2	22	13	12	5	17
MSPL10-6	53,646	34.3	14	2	23	10	9	4	13
TWH P74 ^a^	53,436	34.4	14	2	23	10	9	5	14

^a^ Type culture of *Diaporthe longicolla* (syn. *Phomopsis longicolla*) from American Type Culture Collection (ATCC 60325).

**Table 3:** Number of introns identified from the mitochondrial genome of *Diaporthe longicolla* (syn. *Phomopsis longicolla*) isolates.

Isolate	*atp6*	*cob*	*cox1*	*cox2*	*cox3*	*nad1*	*nad2*	*nad5*	*rnl*	Total
PL1	1	0	4	1	0	1	0	1	1	9
PL6	1	0	4	1	0	1	0	1	1	9
PL7	1	1	5	1	1	1	1	1	1	13
PL10	1	1	4	1	1	1	1	1	1	12
PL11	1	1	4	1	0	1	1	1	1	11
PL185E	1	1	5	1	1	1	1	1	1	13
MSPL10-6	1	1	4	1	0	1	0	1	1	10
TWH P74 ^a^	1	0	5	1	0	1	0	1	1	10

^a^ Type culture from American Type Culture Collection (ATCC 60325).

**Table 4:** Information of introns identified in the mitochondrial genome of *Diaporthe longicolla* isolates.

Gene	Intron	Position	Intron Size (bp)	Intron Type	ORF	Conserved Domain	E-Value	Identity (%)	Similarity	Accession
*cox1*	Intron 1	33 aa ^a^	1406	IB	orf343	GIY-YIG	1 × 10^−152^	76	*Tuber melanosporum Mel28*	XP_002838532.1
	Intron 2	198 aa	1373	-	orf305	LAGLIDADG_1	1 × 10^−172^	86	*Chrysoporthe cubensis*	YP_009262145.1
	Intron 3	206 aa	1080	IB	orf328	double LAGLIDADG_1	0	88	*Candida oxycetoniae*	YP_008475039.1
	Intron 4	251 aa	1134	IB	orf349	LAGLIDADG_1	0	88	*Fusarium graminearum*	YP_001249331.1
	Intron 5	411 aa	1340	IB	orf441	GIY-YIG	0	95	*Sordaria macrospora k-hell*	XP_003342392.1
*cox2*	Intron 1	385 aa	1304	IB(3′)	orf317	GIY-YIG	4 × 10^−144^	66	*Cryphonectria parasitica*	AMX22275.1
*nad5*	Intron 1	239 aa	1039	IB	orf312	LAGLIDADG	1 × 10^−144^	71	*Chrysoporthe austroafricana*	YP_009262028.1
*atp6*	Intron 1	193 aa	1644	IC2	orf295	GIY-YIG	2 × 10^−148^	88	*Podospora anserina*	NP_074919.1
*rnl*	Intron 1	3209 bp	2116	IA	orf516	rps3 gene	0	62	*Chrysoporthe deuterocubensis*	YP_009262125.1
*nad1*	Intron 1	212 aa	1201	IB	orf110	GIY-YIG	2 × 10^−102^	81	*Neurospora crassa OR74A*	YP_009126720.1
*cob*	Intron 1	163 aa	1316	IA	orf315	LAGLIDADG	3 × 10^−179^	85	*Neurospora crassa OR74A*	YP_009126715.1
*nad2*	Intron 1	551 aa	1814	IA	- ^b^	double LAGLIDADG_1	1 × 10^−178^	77	*Chrysoporthe austroafricana*	YP_009262006.1
*cox3*	Intron 1	141 aa	1622	IA	orf390	double LAGLIDADG_1	3 × 10^−133^	68	*Annulohypoxylon stygium*	YP_008964946.1

^a^ Amino acid; ^b^ Values that were not present or not observed.

**Table 5:** Single-nucleotide polymorphism (SNP) position and alleles identified among the mitochondrial genomes of *Diaporthe longicolla* isolates.

			Isolates of *Diaporthe longicolla*						
SNP Position in TWH P74 ^a^	SNP Type	TWH P74	MSPL10-6	PL1	PL185E	PL6	PL10	PL7	PL11
1433	G/T		G	.	T	T	T	T	T
8256	T/G	G	T	G	G	G	G	G	G
8732	A/C	C	A	C	C	C	C	.	C
10,944	A/T	.	A	.	T	.	.	T	.
11,734	G/T	.	G	.	T	.	.	T	.
11,735	T/G	.	T	.	G	.	.	G	.
11,736	A/G	G	A	G	.	.	.	.	.
11,747	A/T	.	A	.	.	.	T	.	.
11,747	A/T	T	A	T	.	.	T	.	.
20,327	C/G	.	G	.	C	C	.	C	C
22,989	T/A	A	T	A	A	A	A	A	A
27,771	G/C	.	G	.	C	.	C	C	.
29,383	G/C	C	G	.	.	.	.	.	.
32,866	G/C	.	G	.	C	C	.	C	C
33,430	G/T	.	G	.	.	.	.	T	.
33,447	G/T	.	G	.	T	.	.	T	.
33,448	T/C	.	T	.	C	.	.	C	.
34,426	A/G	.	A	.	.	.	.	G	.
34,427	A/G	.	A	.	.	.	.	G	.
35,718	C/T	T	C	T	.	.	T	.	.
35,907	A/C	.	A	.	C	C	.	C	C
37,809	A/T	T	A	.	.	T	T	.	T
37,809	C/T	T	C	.	.	T	T	.	T
38,229	T/C	C	T	C	C	C	C	C	C
39,838	T/G	G	T	G	G	G	G	.	G
40,164	C/T	T	C	T	T	T	T	T	T
40,166	G/T	T	G	T	T	T	T	T	T
40,170	A/C	C	A	C	C	C	C	C	C
40,198	T/G	G	T	G	G	G	G	G	G
40,960	A/C	C	A	C	C	C	C	C	C
41,001	T/G	G	T	G	G	G	G	.	G
44,675	A/T	.	A	.	T	.	.	T	.
44,676	T/G	.	T	.	G	.	.	G	.
47,659	T/A	A	T	A	A	A	A	A	A
48,512	G/T	.	G	.	.	.	.	T	.
48,513	A/G	.	A	.	.	.	.	G	.
49,744	T/G	G	T	G	G	.	G	G	.
49,752	A/G	G	A	G	G	.	G	G	.
49,753	T/C	C	T	C	C	.	C	C	.
51,544	T/C	.	T	.	C	C	.	C	C
51,948	T/A	A	T	A	.	A	.	.	.
51,949	G/A	A	G	A	.	A	.	.	.
51,958	A/C	C	A	C	.	C	.	.	.
51,960	A/G	G	A	G	.	G	.	.	.
52,745	A/T	T	A	T	T	T	T	T	T
52,760	A/T	.	A	.	T	.	.	.	.
52,774	T/C	.	T	.	C	.	.	.	.
52,776	T/C	.	T	.	C	.	.	.	.

^a^ The SNPs among the mitochondrial genome sequences of *Diaporthe longicolla* isolates were identified using the DNASP6 program [35], and the SNP positions on the type strain TWH P74 (ATCC 60325) were identified by blasting the flanking sequence to the TWH P74 mitochondrial genome sequence.

## Data Availability

Relevant data generated or analyzed during this study are included in this article. The mitochondrial genome sequences of *D. longicolla* isolates have been deposited in the NCBI GenBank database under the BioProject PRJNA1080574.

## Supplementary Materials

The following supporting information can be downloaded at: https://www.mdpi.com/article/10.3390/jof10080570/s1, Table S1. Local BLASTn analysis of the *Diaporthe longicolla* mitochondrial genome sequences against itself; Table S2. Tandem repeats detected in the mitochondrial genome of *Diaporthe longicolla* using Tandem Repeats Finder; Table S3. Number of repeats in the mitochondrial genome of *Diaporthe longicolla* searched by REPuter; Table S4. Summary of inverted repeats identified by EMBOSS (v6.6.0).

## References

[1] Hobbs T.W., Schmitthenner A.F., Kuter G.A. (1985). A new *Phomopsis* species from soybean. Mycologia.

[2] Hepperly P.R., Sinclair J.B. (1978). Quality losses in *Phomopsis*—Infected soybean seeds. Phytopathology.

[3] Li S., Sudaric A. (2011). Phomopsis seed decay of soybean. Soybean—Molecular Aspects of Breeding.

[4] Li S., Hartman G.L., Boykin D. (2010). Aggressiveness of *Phomopsis longicolla* and other *Phomopsis* spp. on soybean. Plant Dis..

[5] Li S., Chen P., Hartman G.L., Hartman G.L., Rupe C., Sikora J., Domier L.L., Davis J.A., Steffey K.L. (2015). Phomopsis seed decay. Compendium of Soybean Diseases and Pests.

[6] Santos J.M., Vrandecic K., Cosic J., Duvnjak T., Phillips A.J.L. (2011). Resolving the *Diaporthe* species occurring on soybean in Croatia. Persoonia.

[7] Kulik M.M., Sinclair J.B., Hartman G.L., Sinclair J.B., Rupe J.C. (1999). Phomopsis Seed Decay. Compendium of Soybean Diseases.

[8] Sinclair J.B. (1993). *Phomopsis* seed decay of soybeans—A prototype for studying seed disease. Plant Dis..

[9] Minor H.C., Brown E.A., Zimmerman M.S. (1995). Developing soybean varieties with genetic resistance to *Phomopsis* spp.. J. Am. Oil Chem. Soc..

[10] Li S., Rupe J., Chen P., Shannon G., Wrather A., Boykin D. (2015). Evaluation of diverse soybean germplasm for resistance to Phomopsis seed decay. Plant Dis..

[11] Jackson E.W., Fenn P., Chen P. (2005). Inheritance of resistance to Phomopsis seed decay in soybean PI 80837 and MO/PSD-0259 (PI 562694). Crop Sci..

[12] Pathan M.S., Clark K.M., Wrather J.A., Sciumbato G.L., Shannon J.G., Nguyen H.T., Sleper D.A. (2009). Registration of soybean germplasm SS93-6012 and SS93-6181 resistant to Phomopsis seed decay. J. Plant Regist..

[13] Zimmerman M.S., Minor H.C. (1993). Inheritance of Phomopsis seed decay resistance in soybean PI 417479. Crop Sci..

[14] Li S., Sciumbato G., Rupe J., Shannon G., Chen P., Boykin D. (2017). Evaluation of commercial soybean cultivars for reaction to Phomopsis seed decay. Plant Dis..

[15] Li S., Smith R., Zhang L. (2023). Evaluation of exotic soybean accessions and their use in developing improved soybean lines with resistance to Phomopsis seed decay. PLoS ONE.

[16] Li S., Bradley C.A., Hartman G.L., Pedersen W.L. (2001). First report of *Phomopsis longicolla* from velvetleaf causing stem lesions on inoculated soybean and velvetleaf plants. Plant Dis..

[17] Li S. (2018). Development of a seedling inoculation technique for rapid evaluation of soybean for resistance to *Phomopsis longicolla* under controlled conditions. Plant Methods.

[18] Li S., Darwish O., Alkharouf N., Musungu B., Matthews B.F. (2017). Analysis of the genome sequence of *Phomopsis longicolla*: A fungal pathogen causing Phomopsis seed decay in soybean. BMC Genom..

[19] Li S., Deng Y. (2021). Mitochondrial genome resource of *Phomopsis longicolla*, a fungus causing Phomopsis seed decay in soybean. PhytoFrontiers.

[20] Burger G., Gray M.W., Lang B.F. (2003). Mitochondrial genomes: Anything goes. Trends Genet..

[21] Avise J.C., Arnold J., Ball R.M., Bermingham E. (1987). Intraspecific phylogeography: The mitochondrial DNA bridge between population genetics and systematics. Annu. Rev. Ecol. Syst..

[22] Deng Y., Zhang Q., Ming R., Lin L., Lin X., Lin Y., Li X., Xie B., Wen Z. (2016). Analysis of the Mitochondrial genome in *Hypomyces aurantius* reveals a novel twintron complex in fungi. Int. J. Mol. Sci..

[23] Castanera R., López-Varas L., Borgognone A., LaButti K., Lapidus A., Schmutz J., Grimwood J., Pérez G., Pisabarro A.G., Grigoriev I.V. (2016). Transposable Elements versus the Fungal Genome: Impact on Whole-Genome Architecture and Transcriptional Profiles. PLoS Genet..

[24] Bankevich A., Nurk S., Antipov D., Gurevich A.A., Dvorkin M., Kulikov A.S., Kulikov A.S., Lesin V.M., Nikolenko S.I., Pham S. (2012). SPAdes: A New Genome Assembly Algorithm and Its Applications to Single-Cell Sequencing. J. Comput. Biol..

[25] Altschul S.F., Gish W., Miller W., Myers E.W., Lipman D.J. (1990). Basic local alignment search tool. J. Mol. Biol..

[26] Katoh K., Standley D.M. (2013). MAFFT multiple sequence alignment software version 7: Improvements in performance and usability. Mol. Bio. Evol..

[27] Hall T.A. (1999). BioEdit: A user-friendly biological sequence alignment editor and analysis program for Windows 95/98/NT. Nucl. Acids. Symp. Ser..

[28] Camacho C., Coulouris G., Avagyan V., Ma N., Papadopoulos J., Bealer K., Madden T.L. (2008). BLAST+: Architecture and applications. BMC Bioinform..

[29] Benson G. (1999). Tandem repeats finder: A program to analyze DNA sequences. Nucleic Acids Res..

[30] Kurtz S., Choudhuri J.V., Ohlebusch E., Schleiermacher C., Stoye J., Giegerich R. (2001). REPuter: The manifold applications of repeat analysis on a genomic scale. Nucleic Acids Res..

[31] Rice P., Longden I., Bleasby A. (2000). EMBOSS: The European Molecular Biology Open Software Suite. Trends Genet..

[32] Thiel T., Michalek W., Varshney R.K., Graner A. (2003). Exploiting EST databases for the development and characterization of gene-derived SSR-markers in barley (*Hordeum vulgare* L.). Theor. Appl. Genet..

[33] Edgar R.C. (2004). MUSCLE: Multiple sequence alignment with high accuracy and high throughput. Nucleic Acids Res..

[34] Tamura K., Stecher G., Peterson D., Filipski A., Kumar S. (2013). MEGA6: Molecular Evolutionary Genetics Analysis version 6.0. Mol. Biol. Evol..

[35] Rozas J., Ferrer-Mata A., Sánchez-DelBarrio J.C., Guirao-Rico S., Librado P., Ramos-Onsins S.E., Sánchez-Gracia A. (2017). DnaSP 6: DNA sequence polymorphism analysis of large data sets. Mol. Biol. Evol..

[36] Hwang E.Y., Wei H., Schroeder S.G., Fickus E.W., Quigley C.V., Elia P., Araya S., Dong F., Costa L., Ferreira M.E. (2019). Genetic diversity and phylogenetic relationships of annual and perennial Glycine species. G3 Genes Genomes Genet..

[37] Stone C.L., Frederick R.D., Tooley P.W., Luster D.G., Campos B., Winegar R.A., Melcher U., Fletcher J., Blagden T. (2018). Annotation and analysis of the mitochondrial genome of *Coniothyrium glycines*, causal agent of red leaf blotch of soybean, reveals an abundance of homing endonucleases. PLoS ONE.

[38] Stone C.L., Buitrago M.L.P., Boore J.L., Reid D., Frederick R.D. (2010). Analysis of the complete mitochondrial genome sequences of the soybean rust pathogens *Phakopsora pachyrhizi* and *P. meibomiae*. Mycologia.

[39] Mardanov A.V., Beletsky A.V., Kadnikov V.V., Ignatov A.N., Ravin N.V. (2014). The 203 kb Mitochondrial genome of the phytopathogenic fungus sclerotinia borealis reveals multiple invasions of introns and genomic duplications. PLoS ONE.

[40] Salavirta H., Oksanen I., Kuuskeri J., Mäkelä M., Laine P., Paulin L., Lundell T. (2014). Mitochondrial genome of *Phlebia radiata* is the second largest (156 kbp) among fungi and features signs of genome flexibility and recent recombination events. PLoS ONE.

[41] Fonseca P.L., De-Paula R.B., Araújo D.S., Tomé L.M.R., Mendes-Pereira T., Rodrigues W.F.C., Del-Bem L.E., Aguiar E.R., Góes-Neto A. (2021). Global characterization of fungal mitogenomes: New insights on genomic diversity and dynamism of coding genes and accessory elements. Front. Microbiol..

[42] Pramateftaki P.V., Kouvelis V.N., Lanaridis P., Typas M.A. (2006). The mitochondrial genome of the wine yeast *Hanseniaspora uvarum*: A unique genome organization among yeast/fungal counterparts. FEMS Yeast Res..

[43] Liu W., Cai Y., Zhang Q., Chena L., Shu F., Ma X., Bian Y. (2020). The mitochondrial genome of *Morchella importuna* (272.2 kb) is the largest among fungi and contains numerous introns, mitochondrial non-conserved open reading frames and repetitive sequences. Int. J. Bio. Macromol..

[44] Joardar V., Abrams N.F., Hostetler J., Paukstelis P.J., Pakala S., Pakala S.B., Zafar N., Abolude O.O., Payne G., Andrianopoulos A. (2012). Sequencing of mitochondrial genomes of nine *Aspergillus* and *Penicillium* species identifies mobile introns and accessory genes as main sources of genome size variability. BMC Genom..

[45] Wang X., Jia L., Wang M., Yang H., Chen M., Li X., Liu H., Li Q., Liu N. (2020). The complete mitochondrial genome of medicinal fungus *Taiwanofungus camphoratus* reveals gene rearrangements and intron dynamics of *Polyporales*. Sci. Rep..

[46] Lang B.F., Laforest M., Burger G. (2007). Mitochondrial introns: A critical view. Trends Genet..

[47] Saldanha R., Mohr G., Belfort M., Lambowitz A.M. (1993). Group I and group II introns. FASEB J..

[48] Nielsen H., Johansen S.D. (2009). Group I introns: Moving in new directions. RNA Biol..

[49] Ferandon C., Moukha S., Callac P., Benedetto J.P., Castroviejo M., Barroso G. (2010). The Agaricus bisporus cox1 gene: The longest mitochondrial gene and the largest reservoir of mitochondrial group 1 introns. PLoS ONE.

[50] Brankovics B., Dam P., Rep M., Hoog G.S.D., Lee T.A.V.D., Waalwijk C., Diepeningen A.D. (2017). Mitochondrial genomes reveal recombination in the presumed asexual *Fusarium oxysporum* species complex. BMC Genom..

[51] Aguileta G., de Vienne D.M., Ross O.N., Hood M.E., Giraud T., Petit E., Gabaldon T. (2014). High variability of mitochondrial gene order among fungi. Genome Biol. Evol..

[52] Li Y., Hu X.-D., Yang R.-H., Hsiang T., Wang K., Liang D.-Q., Wang K., Liang F., Cao D.-M., Zhou F. (2015). Complete mitochondrial genome of the medicinal fungus *Ophiocordyceps sinensis*. Sci. Rep..

[53] Li Q., Chen C., Xiong C., Jin X., Chen Z., Huang W. (2018). Comparative mitogenomics reveals large-scale gene rearrangements in the mitochondrial genome of two *Pleurotus* species. Appl. Microbiol. Biotechnol..

[54] Lin R., Liu C., Shen B., Bai M., Ling J., Chen G., Mao Z., Cheng X., Xie B. (2015). Analysis of the complete mitochondrial genome of *Pochonia chlamydosporia* suggests a close relationship to the invertebrate-pathogenic fungi in Hypocreales. BMC Microbiol..

[55] Jelen V., Jonge R.D., Peer Y.V.D., Jakše J.J.B. (2016). Complete mitochondrial genome of the Verticillium-wilt causing plant pathogen *Verticillium nonalfalfae*. PLoS ONE.

[56] Wostemeyer J., Kreibich A. (2002). Repetitive DNA elements in fungi (Mycota): Impact on genomic architecture and evolution. Curr. Genet..

[57] Losada L., Pakala S.B., Fedorova N.D., Joardar V., Hostetler S.A.S.J., Pakala S.M., Zafar N., Thomas E., Rodriguez-Carres M., Dean R. (2014). Mobile elements and mitochondrial genome expansion in the soil fungus and potato pathogen *Rhizoctonia solani* AG-3. FEMS Microbiol. Lett..

[58] Mahfooz S., Singh P., Maurya D.K., Yadav M.C., Tahoor A., Sahay H., Srivastava A.A., Prakash A. (2012). Microsatellite repeat dynamics in mitochondrial genomes of phytopathogenic fungi: Frequency and distribution in the genic and intergenic regions. Bioinformation.

[59] Eo J.-K. (2021). The complete mitogenome of *Diaporthe nobilis*. Mitochondrial DNA Part B.

[60] Sandor S., Zhang Y., Xu J. (2018). Fungal mitochondrial genomes and genetic polymorphisms. Appl. Microbio. Biotechnol..

[61] Wang Y., Xu J. (2020). Mitochondrial genome polymorphisms in the human pathogenic fungus *Cryptococcus neoformans*. Front. Microbiol..

[62] Ye F., Yu X.-D., Zhao P. (2016). Identification of SNPs in a nonmodel macrofungus (*Lepista nuda*, Basidiomycota) through RAD sequencing. SpringerPlus.

[63] Tan M.-K., Raman H., Chambers G., Sharma I., Chen Z., Deshpande N., Wilkins M.R. (2016). Characterization of SNP and structural variations in the mitochondrial genomes of *Tilletia indica* and its closely related species formed basis for a simple diagnostic assay. PLoS ONE.

[64] Kulik T., Brankovics B., van Diepeningen A.D., Bilska K., Zelechowski M., Myszczynski K., Molcan T., Stakheev A., Stenglein S., Beyer M. (2020). Diversity of Mobile GeneticElements in the Mitogenomes of Closely Related *Fusarium culmorum* and *F. graminearum* sensu stricto Strains and Its Implication. Front. Microbiol..

[65] Wyrebek J., Molcan T., Myszczynski K., van Diepeningen A.D., Stakheev A.A., Zelechowski M., Bilskan K., Kulik T. (2021). Uncovering diagnostic value of mitogenome for identification of cryptic species *Fusarium graminearum* sensu stricto. Front. Microbiol..

[66] Agrios G.N. (2005). Plant Pathology.

