# Supplementary material for: Comparative Analysis of the Mitochondrial Genome Sequences of Diaporthe longicolla (syn. Phomopsis longicolla) Isolates Causing Phomopsis Seed Decay in Soybean
Source: J Fungi (Basel). 2024 Aug 13;10(8):570. doi: 10.3390/jof10080570 (PMC11355892; doi:10.3390/jof10080570)
Supplement: Supplementary file 1 [file jof-10-00570-s001.zip › jof-2913112-supplementary.pdf]

Table S1. Local BLAST analysis of the *Diaporthe longicolla* mitochondrial genome against itself.

Isolate	Identity range	Aligned length range	E-value range
PL1	90 ~100 %	33 ~ 336	4.87E-120 ~ 5.69E-10
PL6	90 ~100 %	34 ~ 336	1.10E-121 ~ 5.69E-10
PL7	88 ~100 %	34 ~ 336	1.30E-121 ~ 7.06E-10
PL10	90 ~100 %	34 ~ 336	5.80E-120 ~ 6.77E-10
PL11	90 ~100 %	34 ~ 336	1.20E-121 ~ 6.39E-10
PL185E	90 ~100 %	34 ~ 336	1.30E-121 ~ 7.05E-10
MSPL10-6	85 ~100 %	34 ~ 336	5.10E-120 ~ 5.98E-10
TWH P74	90 ~100 %	34 ~ 336	5.10E-120 ~ 5.94E-10

Table S2. Tandem repeats detected in the mitochondrial genome of *Diaporthe longicolla* using Tandem Repeats Finder.

Repeat motif	PL1	PL6	PL7	PL10	PL11	PL1 85E	MSPL 10-6	TWH P74
CCTTAGCAAAGCCAGGCC GGCTTCT	+	+	+	+	+	+	+	+
TAAGATAAAATCCGGTAT GAATACAT	-	-	+	+	+	+	-	-
ATTTATTTTATTTGTTAGT ACTAA	+	+	+	+	+	+	+	+
AAAGCATTAACTAAATTA AGTA	+	+	+	+	+	+	+	+
TAATTGGTACAGGTT	+	+	+	+	+	+	+	+
AGACGTAGTCGGC	+	+	+	+	+	+	+	+
CGCACTCCTTCCTCCTACG AAGTCAGG CAGGAGGCTT	+	+	+	+	+	+	+	+
GGCTACGC	+	+	+	+	+	+	+	+

Table S3. Number of repeats in the mitochondrial genomes of *Diaporthe longicolla* searched by REPuter.

Isolate	Forward match	Palindromic match	Reverse match	Complement match
PL1	31	19	0	0
PL6	31	19	0	0
PL7	30	20	0	0
PL10	31	19	0	0
PL11	31	19	0	0
PL185E	31	19	0	0
MSPL10-6	31	19	0	0
TWH P74	31	19	0	0

Table S4. Summary of inverted repeats identified by EMBOSS (v6.6.0).

Isolate	Number of short length inverted repeats	Size range (bp)	Total length	Percentage of the genome
MSPL 10-6	22	17-50	1185	2.21
TWH 74	20	17-54	1060	1.98
PL185E	22	17-54	1149	1.97
PL1	21	17-54	1104	2.10
PL6	20	17-54	1061	2.03
PL7	21	17-48	1091	1.87
PL10	22	17-54	1155	2.02
PL11	21	17-54	1112	1.99
